# The Institutional Worker

**Published:** 1912-06-01

**Authors:** 


### INSTITUTIONAL WORKER

**Being a Special Supplement to "The Hospital"**

### Editor's Notices.

**Contributions:**

Contributions should be written, or preferably typed, on one side of the paper only, and all articles sent in are accepted upon the distinct understanding that they are forwarded to THE HOSPITAL only.

The Editor cannot undertake to return MSS. not used, but when a stamped directed envelope is enclosed the MSS. may be returned if a special request is made.

Accepted articles and paragraphs of news will be paid for after publication at the scale rate.

**Address:**

To prevent delay all contributions and letters on editorial business must be addressed exclusively to the Editor, "The Hospital," 29 Southampton Street, Strand, London, W.C. It is important that this regulation shall be strictly observed.

**Correspondence:**

Correspondence on all subjects is invited. The name and address of correspondents must be given as a guarantee of good faith, but not necessarily for publication.

**Special Articles:**

Special articles are invited, and questions, inquiries, and paragraphs upon all matters relating to the work, administration, and management of general, special, mental, fever, cottage and convalescent hospitals, sanatoria, homes, institutions, societies and organisations for the treatment or care of the sick, injured and dependants of all classes. Every matter affecting the interests and welfare of the staff of all grades working in these institutions will receive special consideration.

**Administrative Medicine, Research and Items of News:**

Special terms will be paid for approved articles on subjects in this wide field by experts who have made some section of it their special study and interest. We wish to give prominence to every movement tending to advance accuracy of diagnosis, efficiency in treatment, and the development of modern methods to eradicate disease and promote the welfare of the suffering.

**A Bureau of Information:**

We invite inquiries and applications for information and help in providing for that numerous class of sufferers who require special care or house-room which they cannot obtain in their own homes or secure for themselves. **We cannot, however, prescribe, or recommend practitioners.**

**Books for Review:**

Publishers are particularly requested to send advance proofs of any new books of importance whenever possible, as the Editor has made arrangements to publish immediate reviews on a new plan.

**Photographs, Plans, Blocks, and Illustrations:**

It is requested that wherever possible MSS. may be accompanied by illustrations in any of the above forms. The name of the sender and of the article to which it belongs should be written on the back of each photograph, drawing, or block for purposes of identification.

**Local Papers:**

Newspapers containing reports or news paragraphs **should be marked** and addressed to the Sub-Editor.

**The Coupon System:**

A coupon will be found at the bottom of the third inside page of the cover each week. This coupon must be attached to every question or inquiry to which an answer is desired in THE HOSPITAL.

### An Appointment Wanted.

The purpose of THE INSTITUTIONAL WORKER primarily is to be of the utmost assistance to all seeking appointments in any branch of the Institution, Health, and Sanitary services. A large number of announcements of vacancies appear in its pages every week, and it is our object to render this section of THE HOSPITAL of the greatest practical utility to everyone engaged in or attracted to Institutional Work. In furtherance of this aim, a special column is reserved for Appointments Wanted, in which you may give effective publicity to your needs for the small sum of one shilling for twenty-one words. Through the medium of this column it is possible to bring your requirements and capabilities prominently before the Administrative Officials of Institutions of every description throughout the United Kingdom.

The form printed below is for the convenience of our readers who wish to take advantage of the exceptional facilities which THE INSTITUTIONAL WORKER affords, and when filled up should be posted with remittance to

*The Manager*, THE HOSPITAL,
28 and 29 Southampton Street,
Strand, W.C.

Appointments Wanted, 21 words ... 1s. 0d.

| | | |
|---|---|---|
| | | |
| | | |
| | | |
| | | |
| | | |
| | | |

### Manager's Notices.

**All letters on business matters must be addressed to The Manager, "The Hospital," 28 and 29 Southampton Street, London, W.C.**

**Subscriptions** may begin at any time, and are payable in advance. Cheques and Post-Office Orders should be crossed London County and Westminster Bank, Covent Garden Branch, and made payable to the Manager of THE HOSPITAL.

RATES OF SUBSCRIPTION (payable in advance).

UNITED KINGDOM.

| | | |
|---|---|---|
| Three Months (including Postage) | ... ... ... ... | 2s. 0d. |
| Six Months | do. ... ... ... ... | 4s. 0d. |
| Twelve Months | do. ... ... ... ... | 6s. 6d. |

FOREIGN AND COLONIAL.

| | | |
|---|---|---|
| Three Months (including Postage) | ... ... ... ... | 2s. 6d. |
| Six Months | do. ... ... ... ... | 5s. 0d. |
| Twelve Months | do. ... ... ... ... | 8s. 8d. |

**Advertisements:**

To ensure insertion, all advertisements for THE HOSPITAL must reach the Manager not later than Wednesday morning in each week. For scale of charges see pages vii and 2.

**Remittances:**

**It is especially requested that remittances be made by Cheque or Postal Order, and in halfpenny stamps only when the amount is under sixpence.**

### OFFICIAL APPOINTMENTS VACANT.

### Poor-Law Vacancies.

Assistant Engineer (£60), Willesden Guardians. Mr. J. H. Haylor, C. to G., 357 High Road, Brondesbury, N.W. June 5.

Assistant Master (£70), West Ham Union. Mr. T. Smith, C. to G., Leytonstone, N.E. June 5.

Assistant Nurse (£28), Eastry Union. Mr. F. S. Cloke, C. to G., Sandwich. June 3.

Assistant Nurses (£24), Watford Union. Mr. F. Wilson, C. to G., Watford. June 4.

Assistant Nurse (£22), Ormskirk Union. Mr. A. Dickinson, C. to G., Ormskirk. June 5.

Assistant Nurse (£25), Solihull Union. Mr. F. L. Thompson, C. to G., Sparkhill, Birmingham. June 6.

Assistant Lunacy Attendant (£32), Stoke-on-Trent Union. Mr. T. Wood, C. to G., Stoke-on-Trent. June 10.

Barber, etc. (£30), Ormskirk Union. Mr. A. Dickinson, Ormskirk. June 5.

Barber and Bath Attendant (£30), Stoke-on-Trent Union. Mr. T. Wood, C. to G., Stoke-on-Trent. June 10.

Charge Nurse (£30), Cardiff Union. Mr. A. J. Harris, C. to G., Cardiff. June 3.

Charge Nurse (£32), Watford Union. Mr. F. Wilson, C. to G., Watford. June 4.

Charge Nurse (£30), Rochford Union. Mr. F. Gregson, C. to G., Southend-on-Sea. June 10.

Charge Nurse (£35), Hull Guardians. R. H. Winter, C. to G., St. Mary's Chambers, Lowgate, Hull. June 4.

Checker and Laundry Assistant (£20), Middlesbrough Union. Mr. J. J. Dales, C. to G., Middlesbrough. June 5.

Collector of Rates (£95), Neath Union. Mr. H. Cuthbertson, C. to G., Neath. June 3.

Cook (£20), Chepstow Union. Mr. F. Evans, C. to G., Chepstow. June 6.

Female Attendants (£30), Reading Guardians. Mr. W. H. Oliver, C. to G., Reading. June 12.

Female Assistant Relieving Officer (£80), Leicester Guardians. Mr. H. Mansfield, C. to G., Leicester. June 10.

Female Lunatic Attendant (£22), Cardiff Union. Mr. A. J. Harris, C. to G., Cardiff. June 3.

Female Imbecile Attendant (£25), Keighley Union. Mr. S. Green, C. to G., Keighley. June 3.

Female Infirm Attendant (£25), Wigan Union. Mr. H. G. Ackerley, C. to G., Wigan. June 5.

Female Mental Nurse (£25), Hartlepool Union. Mr. G. Kilvington, C. to G., West Hartlepool. June 11.

Female Ward Attendant (£20), Sheppey Union. Mr. J. Copland, C. to G., Sheerness. June 4.

Foster-Mother (£25), Belper Union. Mr. J. Pym, C. to G., Belper. June 7.

Laundress (£27 10s.), Thetford Union. Mr. J. Houchen, C. to G., Thetford, Norfolk. June 6.

Lady Clerk (27s. 6d. weekly, non-resident), West London School District. Mr. F. G. Beeching, Clerk to the Managers, Ashford, Middlesex. June 3.

Male Attendant (£30), Ormskirk Union. Mr. A. Dickinson, C. to G., Ormskirk. June 5.

Male Cook (£35), Blackburn Union. Mr. C. E. Bygrave, C. to G., Blackburn. June 4.

Male General Assistant (£32 5s.), Leigh Union. Mr. A. H. Hayward, C. to G., Leigh. June 5.

Male Imbecile Attendant (£26), Barton-upon-Irwell Union. Mr. J. W. Whitworth, C. to G., Patricroft, Manchester. June 4.

Male Imbecile Attendant (£30), Burnley Union. Mr. J. S. Horn, C. to G., Burnley. June 5.

Master and Matron (£120 and £60), Stockport Union. Mr. C. F. Johnson, C. to G., Stockport. June 10.

Master's Junior Clerk (9s. weekly), Southwark Union. Mr. S. Wood, C. to G., Ufford Street, Blackfriars, S.E. June 4.

Night Nurse (£25), Biggleswade Union. Mr. G. Wagg, C. to G., Biggleswade, Beds. June 5.

Night Sister (£33), Wakefield Union. H. Beaumont, C. to G., 47 Kirkgate, Wakefield. June 4.

Nurse (£30), Ormskirk Union. A. Dickinson, C. to G., Union Offices, Ormskirk. June 5.

Nurse (£32 12s.), Kington Union. Mr. B. Philpin, C. to G., Kington; Hereford. June 3.

Nurse (£28), Halifax Union. Mr. A. T. Longbotham, C. to G., Halifax. June 3.

Nurse (£30), Richmond Union. Mr. P. Umney, C. to G., Richmond, Surrey. June 7.

Nurse (£30), Swindon and Highworth Union. Mr. J. P. Kirby, C. to G., Swindon. June 11.

Nurses (£38), Plympton St. Mary Union. Mr. J. W. Bickle, C. to G., Temple Chambers, Westwell Street, Plymouth. June 6.

Porter and Handy Man (£25), Stockport Union. Mr. C. F. Johnson, C. to G., Stockport. June 6.

### Books for Institutional Workers.

"Midwife's Pocket Dictionary, with revised Rules of the C.M.B." ... 1s. 1d.
"The Nurses' Pronouncing Dictionary" ... 2s. 0d.
"A Handbook for Nurses." (J. K. Watson, M.D.) 5s. 4d.
"Art of Feeding the Invalid." ... 1s. 6d.
"Surgical Ward Work and Nursing." ... 5s. 5d.
"Massage Movements, including the Nauheim Exercises" ... 1s. 1d.
"Surgical Instruments and Appliances used in Operations" (Harold Burrows, M.B.London, B.S., F.R.C.S.) ... 1s. 8d.
"The Nurse's Materia Medica" (Herbert French, M.D.) ... 2s. 9d.

Case-papers, Charts, Professional Cards, and every kind of Institutional Literature.

Of all Booksellers or of The Scientific Press, Limited, 28 and 29 Southampton Street, Strand, London, W.C.

